# A Novel FYVE Domain-Containing Protein Kinase, PsZFPK1, Plays a Critical Role in Vegetative Growth, Sporangium Formation, Oospore Production, and Virulence in *Phytophthora sojae*

**DOI:** 10.3390/jof9070709

**Published:** 2023-06-28

**Authors:** Binglu Ru, Xinchang Hao, Wenhao Li, Qin Peng, Jianqiang Miao, Xili Liu

**Affiliations:** 1 State Key Laboratory of Crop Stress Biology for Arid Areas, College of Plant Protection, Northwest A&F University, 3 Taicheng Road, Yangling, Xianyang 712100, China; rubinglu@163.com (B.R.);; 2 Department of Plant Pathology, College of Plant Protection, China Agricultural University, 2 Yuanmingyuanxi Road, Beijing 100193, China

**Keywords:** *Phytophthora sojae*, protein kinase, FYVE domain, phosphorylation site, phenotype

## Abstract

Proteins containing both FYVE and serine/threonine kinase catalytic (STKc) domains are exclusive to protists. However, the biological function of these proteins in oomycetes has rarely been reported. In the *Phytophthora sojae* genome database, we identified five proteins containing FYVE and STKc domains, which we named PsZFPK1, PsZFPK2, PsZFPK3, PsZFPK4, and PsZFPK5. In this study, we characterized the biological function of PsZFPK1 using a CRISPR/Cas9-mediated gene replacement system. Compared with the wild-type strain, P6497, the *PsZFPK1*-knockout mutants exhibited significantly reduced growth on a nutrient-rich V8 medium, while a more pronounced defect was observed on a nutrient-poor Plich medium. The *PsZFPK1*-knockout mutants also showed a significant increase in sporangium production. Furthermore, PsZFPK1 was found to be essential for oospore production and complete virulence but dispensable for the stress response in *P. sojae*. The N-terminal region, FYVE and STKc domains, and T602 phosphorylation site were found to be vital for the function of PsZFPK1. Conversely, these domains were not required for the localization of PsZFPK1 protein in the cytoplasm. Our results demonstrate that PsZFPK1 plays a critical role in vegetative growth, sporangium formation, oospore production, and virulence in *P. sojae*.

## 1. Introduction

Oomycetes, such as *Phytophthora* spp., encompass a wide variety of plant pathogens that cause devastating diseases in various crops [1]. The economic impacts of root and stem rots caused by *Phytophthora sojae* have resulted in it being ranked among the 10 most destructive and impactful oomycete pathogens [2,3]. *P. sojae* was the first oomycete to have its genome sequenced, and its transcriptome data are also available [4,5]. Because of its ease of culture and simple genetic operability, this species has become a model for studying plant and pathogen interactions and functional genomics. In particular, the establishment of the CRISPR/Cas9-mediated gene knockout system has greatly advanced research into the functional genomics of *P. sojae* [6,7].

The AGC kinase family comprises a group of kinases that are structurally and functionally related. They are named after the cAMP-dependent protein kinase (PKA), cGMP-dependent protein kinase (PKG), and protein kinase C (PKC). These kinases are responsible for the transduction of various extracellular signals and the regulation of growth and development [8,9]. The activation of most AGC protein kinases requires the phosphorylation of two highly conserved phosphorylation sites: one within the activation loop (T-loop) in the catalytic domain and another within the hydrophobic motif (HM) in the C-terminal extension region [8,10]. Functional studies of AGC kinases encompass animals, plants, and pathogenic microbiology. Several studies have demonstrated the importance of AGC kinases in various key processes in plant pathogenic fungi and oomycetes. For example, the protein kinase FgSch9 regulates multiple stress responses and secondary metabolism in *Fusarium graminearum* [11], while the AGC kinase SsAgc1 regulates mating, filamentation, and pathogenicity in *Sporisorium scitamineum* [12]. Another AGC kinase, PgAGC1, regulates mycelial growth and virulence in the entomopathogenic oomycete *Pythium guiyangense* [13]. In addition, the deletion of PsYPK1, an AGC protein kinase found in *P. sojae*, resulted in a pronounced reduction in sporangia and oospore production and virulence [14].

The FYVE domain is a protein domain that specifically binds to phosphatidylinositol-3-phosphate (PI3P). Its name is derived from the initials of the proteins that contain this domain, Fab1p, YOTB, Vac1p, and EEA1 (early endosomal antigen-1) [15,16]. The FYVE domain comprises eight conserved cysteines, as well as an N-terminal WxxD motif, an R(R/K)HHCR motif (the specific binding site for PI3P), and an RVC motif in the C-terminus [17,18]. This domain is widespread in eukaryotes and, interestingly, has been found to be expanded in the *Phytophthora* genome [19]. The number of FYVE proteins in the *P. sojae* genome is 191, which greatly exceeds the number found in humans (27), *Arabidopsis thaliana* (15), or yeast (5) [16,20,21]. FYVE domain-containing proteins play a crucial role in membrane trafficking, cytoskeletal regulation, and receptor signaling through specific binding to PI3P [18]. For instance, the *P. sojae* FYVE protein PsFYVE1 modulates immunity-related gene expression by targeting the host RZ 1A protein [22]. Additionally, the fusion of the antimicrobial peptides GAFP1 or GAFP3 with the FYVE domain enhanced their ability to boost resistance to different *Phytophthora* pathogens [23]. Furthermore, the *P. sojae* FYVE protein PsFP1 is essential for mycelial growth, virulence, and the oxidative stress response [21].

According to Banerjee et al., proteins containing both FYVE and STKc domains are found exclusively in protists [19]. Vassella et al. reported the only instance of a protein kinase containing the FYVE domain in *Trypanosoma brucei*, the deletion of which resulted in an increased differentiation rate [24]. In oomycetes, bioinformatic searches of the kinome of *Phytophthora infestans* revealed the presence of proteins combining FYVE and STKc domains, as reported by Judelson et al. [25]. However, the biological function of these kinases in *Phytophthora* spp. has not yet been investigated. The uncovering of these protein kinase functions will enrich the understanding of functional genes and protein kinases in oomycetes, and will provide important guidance for the discovery of potential fungicide molecular targets.

In this study, we identified five AGC protein kinases with FYVE domains as accessory domains in the genome of *P. sojae*, which we named PsZFPK1, PsZFPK2, PsZFPK3, PsZFPK4, and PsZFPK5. We characterized the role of PsZFPK1 using a CRISPR/Cas9-mediated gene replacement system and found that it plays a critical role in vegetative growth, sporangium formation, oospore production, and virulence in *P. sojae*.

## 2. Materials and Methods

### 2.1. Sequence and Phylogenetic Analyses of PsZFPK1-5

The amino acid sequences of PsZFPK1–5 were obtained from the JGI genome database (https://genome.jgi.doe.gov/portal/, accessed on 20 June 2022) using PITG_01365 as query sequence. To correct the coding sequence, the gene model was verified by reverse transcription-PCR (RT-PCR) using the primers listed in Table S1. Conserved protein domains were analyzed using SMART (http://smart.embl.de/, accessed on 25 June 2022) and Pfam (http://pfam.xfam.org/, accessed on 25 June 2022) search programs. Multiple alignments of the amino acid sequences of FYVE and STKc domains were initially performed using Clustal X [26]. Using PsZFPK1 as the query sequence, homologous protein sequences corresponding to PsZFPK1–5 were retrieved from the Ensembl Protists database (http://protists.ensembl.org/index.html, accessed on 25 June 2022) using BlastP. A phylogenetic tree was constructed using Bayesian inference (BI) by MrBayes v. 3.2.7 with two independent runs, each with four chains for 30,000 generations with sampling every 1000 generations and posterior probabilities (PP) calculated after discarding the first 25% samples [27].

### 2.2. P. sojae Strains and Culture Conditions

*P. sojae* P6497 wild-type strain with known genome sequencing data was used in this study. This strain, along with all transformants, was cultured on 10% V8 medium in darkness at 25 °C. To assess mycelial growth, an agar disc (5 mm agar in diameter) was taken from the edge of an actively growing culture of P6497 or transformant and placed in the center of a V8 plate. Colony diameter was measured after 7 days of growth. Plich medium, a minimal medium with poor nutritional content, was also used for growth assays; per liter, this medium contains 0.5 g KH_2_PO_4_, 0.5 g yeast extract, 1 g asparagine, 0.25 g MgSO_4_·7H_2_O, 1 mg thiamine, 25 g glucose, 10 mg β-sitosterol, and 15 g agar [28].

### 2.3. RNA Extraction and Gene Expression Analysis of PsZFPK1

Materials from different developmental stages of *P. sojae,* including mycelia (MY), sporulating hyphae (SP), zoospores (ZO), cystospores (CY), germinated cystospores (GC), and the process of infection (at 1.5, 3, 6, 12, 24, and 48 h), were collected as described previously [29]. Total RNA was extracted using the SV Total RNA Isolation kit (Promega, Madison, WI, USA) following the recommended protocol, and RNA quality was tested via agarose gel electrophoresis. First-strand cDNA was synthesized from 1 μg of total RNA using the FastKing Reverse Transcriptase kit (Tiangen, Beijing, China). Quantitative real-time PCR (qRT-PCR) was performed on a Bio-Rad CFX Connect Real-Time PCR System using SuperReal qPCR Mix (Tiangen) following the manufacturer’s instructions. Actin sequence from *P. sojae* was used as a reference gene, and the primers for this experiment are listed in Table S1. Relative *PsZFPK1* transcript levels were calculated using the 2^−ΔΔCT^ method [30]. Means and standard deviations were calculated using data from three biological replicates.

### 2.4. Plasmid Construction

Gene knockout transformants were generated using the CRISPR/Cas9-mediated gene replacement strategy, as described by Fang et al. [6]. The sgRNAs were designed using the web tool EuPaGDT (http://grna.ctegd.uga.edu, accessed on 25 June 2022) and cloned into pYF515 plasmids, which contain Cas9 and the selection marker NPTII, following a previously described protocol [7]. The donor vector, pBS-SK II+, contained the sequences 1 kb upstream of *PsZFPK1*, the replacement gene *mCherry*, and 1 kb of *PsZFPK1* downstream sequence. The full-length version of *PsZFPK1*, as well as truncated versions lacking the N-terminus, FYVE domain, or STKc domain, were inserted into the pYF3 vector (containing the Ham34 promoter) to produce GFP fusion proteins for the observation of subcellular localization. The sgRNAs and primers used in this experiment are listed in Table S1.

### 2.5. Generation of P. sojae Transformants

Gene knockout transformants were generated using the CRISPR/Cas9-mediated gene replacement strategy, following a previously described protocol [7]. The transformants were obtained using PEG-mediated protoplast transformation. Positive transformants were screened on V8 plates containing 50 µg/mL G418 and confirmed by PCR and RT-PCR. In situ complementation transformations were carried out using previously described methods [31]. The process for obtaining fluorescent-locatable transformants was similar to that used for gene knockout. Putative transformants were selected on V8 medium containing 50 µg/mL G418 and confirmed using a fluorescence microscope.

### 2.6. Phenotypic Analysis of Transformants

For the analysis of mycelial growth, P6497 and its transformants were cultivated on V8 and Plich medium at 25 °C in the dark. Colony diameters were measured after 7 days on V8 medium and 9 days on Plich medium. Relative colony diameters were then calculated.

For sporangium production, P6497 and its transformants were cultured on V8 plates at 25 °C in the dark for 7 days. The plates were then washed with sterile water eight times at 20 min intervals, followed by incubation at room temperature for 4–6 h. Numbers of sporangia were determined using microscopy at 100× magnification, and relative sporangium numbers were calculated.

For oospore production, P6497 and its transformants were grown on V8 medium in the dark at 25 °C for 10 days. Oospore production was examined by microscopy at 40× magnification, and relative oospore numbers were calculated.

For the analysis of pathogenicity, etiolated seedlings of soybean cultivar (Williams) were cultured for four days in a greenhouse and inoculated with 200 zoospores/10 µL on the hypocotyls. Prior to inoculation, the hypocotyls of etiolated soybean seedlings were scratched using a pipette tip for the wound-inoculation assay. The length of the lesion (browning region) was measured 48 h later, and biomass data were determined using quantitative real-time polymerase chain reaction (qRT-PCR) analysis.

All assays were repeated at least three times. Differences in significance were analyzed using the least significant difference multiple range test at *p* = 0.01.

### 2.7. Subcellular Localization

Full-length and truncated versions of *PsZFPK1* were introduced into P6497 via PEG-mediated protoplast transformation, as described by Fang et al. [7]. The transformants were cultured in 10% liquid V8 medium supplemented with 50 μg/mL G418 for two days and observed using confocal microscopy (Zeiss 900) to detect GFP fluorescence. Transformants exhibiting GFP fluorescence were selected for total protein isolation using a protein extraction kit according to the manufacturer’s instructions (Bestbio, Shanghai, China). The resulting lysates were analyzed by Western blotting using an anti-GFP antibody (Abways, Shanghai, China).

## 3. Results

### 3.1. Sequence and Phylogenetic Analyses of Five Putative PsZFPKs

**Figure 1:**
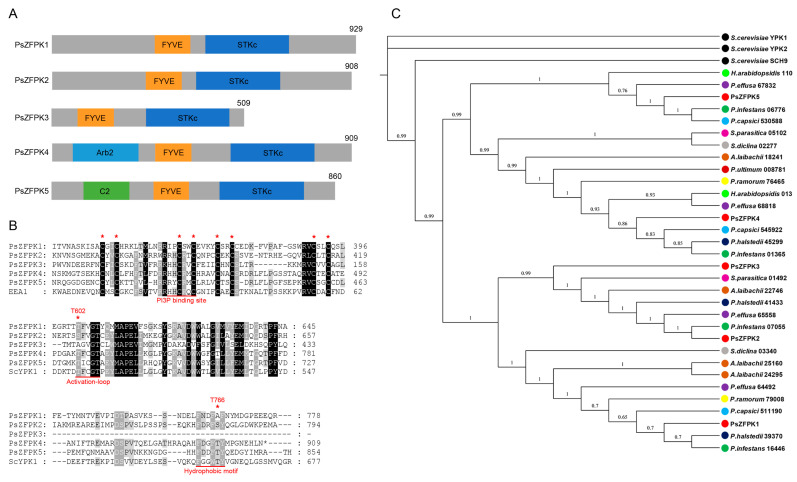
Bioinformatic analysis of five PsZFPKs. (**A**) Full-length PsZFPK proteins and location of the conserved domains. (**B**) Partial amino acid sequence alignment of the PsZFPK proteins, mouse EEA1 (GenBank: AAH75637.1), and *Saccharomyces cerevisiae* YPK1 (GenBank: CAA81967.1); the red asterisk indicates the eight conserved cysteines. (**C**) Phylogenetic tree of PsZFPK proteins and oomycete proteins with FYVE and STKc domains. The Bayesian inference (BI) tree shows PsZFPK proteins and homologs from *Phytophthora capsici* (light blue circle), *Phytophthora infestans* (bottle green circle), *Phytophthora ramorum* (yellow circle), *Pythium ultimum* (crimson circle), *Hyaloperonospora arabidopsidis* (light green circle), *Albugo laibachii* (brown circle), *Peronospora effusa* (purple circle), *Plasmopara halstedii* (dark blue circle), *Saprolegnia diclina* (gray circle), *Saprolegnia parasitica* (pink circle), and *Saccharomyces cerevisiae* (black circle).

Using an FYVE domain-containing protein kinase (PITG_01365) from *P. infestans* as the query sequence, we identified five proteins with FYVE and STKc domains in the genome of *P. sojae*, named PsZFPK1 (345221), PsZFPK2 (354099), PsZFPK3 (501202), PsZFPK4 (360424), and PsZFPK5 (555263) (Figure 1A). Conserved domain prediction for PsZFPK1 confirmed the presence of an N-terminal FYVE domain and C-terminal STKc domain (Figure 1A). However, the amino acid sequence of the predicted PI3P binding site of the FYVE domain in PsZFPK1 was RRIPCS, rather than the typical R(R/K)HHCR motif (Figure 1B). In addition, AGC protein kinases contain two conserved phosphorylation sites in the T-loop and HM region, which correspond to positions 602 and 766 of PsZFPK1; however, we found that the amino acid at site 766 was naturally alanine rather than threonine (Figure 1B). The homologous proteins of PsZFPK1–5 were also identified in other oomycetes, while phylogenetic tree analysis showed that PsZFPK proteins 1–5 were distributed in different clades, indicating that PsZFPKs may be functionally differentiated (Figure 1C).

### 3.2. Expression Pattern Analysis of PsZFPK1

**Figure 2:**
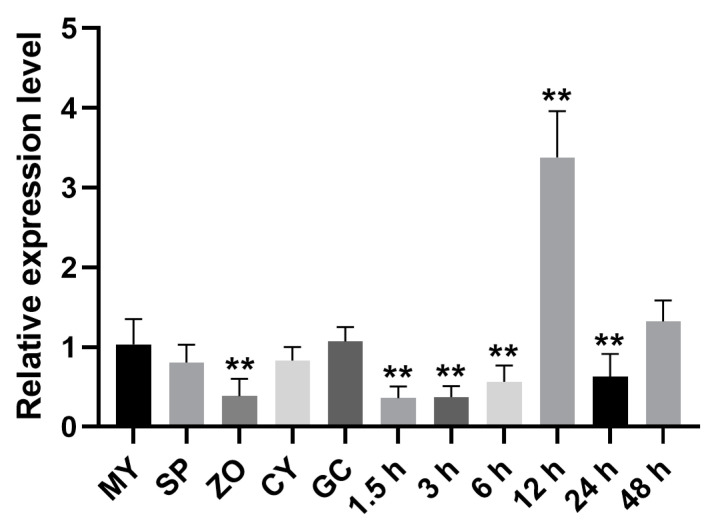
Expression profile of the *PsZFPK1* gene. *PsZFPK1* expression was profiled using quantitative reverse transcription-PCR in materials obtained throughout the *Phytophthora sojae* life cycle, including mycelia (MY), sporulating hyphae (SP), zoospores (ZO), cystospores (CY), and germinated cystospores (GC), and during the process of infection at 1.5, 3, 6, 12, 24, and 48 h post-inoculation (hpi). Relative expression levels were calculated using the MY values as references. A one-way ANOVA was used for statistical analysis, and asterisks indicate significant difference at *p* < 0.01 (**).

The PCR efficiency values for *PsActin* and *PsZFPK1* were 92% and 92.2%, respectively (Figure S1). *PsZFPK1* transcripts were found to be abundant in *P. sojae* at various developmental and infection stages. During infection, at 12 h, transcripts were up-regulated more than three-fold, compared with expression within mycelia (Figure 2), indicating that PsZFPK1 may play a crucial role in the middle stage of infection. Interestingly, *PsZFPK1* expression was found to be decreased during the sporulating hyphae (SP) and zoospore (ZO) stages, suggesting that PsZFPK1 may negatively regulate asexual reproduction (Figure 2).

### 3.3. PsZFPK1 Plays a Critical Role in Vegetative Growth, Sporangium Formation, Oospore Production, and Virulence in P. sojae

**Figure 3:**
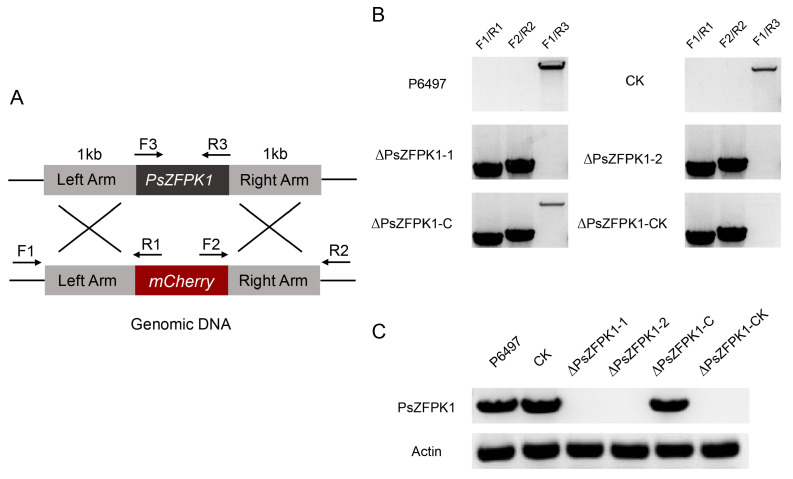
CRISPR/Cas9-mediated *PsZFPK1* gene knockout and complementation. (**A**) Primer locations for transformant screening are indicated by the black arrows. (**B**) PCR results demonstrated that *PsZFPK1* was completely replaced by the *mCherry* gene and successfully complementary in situ. P6497, wild-type; CK, unsuccessful *PsZFPK1* knockout strain; ΔPsZFPK1-1 and ΔPsZFPK1-2, *PsZFPK1* knockout mutants; ΔPsZFPK1-C, *PsZFPK1* complementary strain; and ΔPsZFPK1-CK, unsuccessfully complemented *PsZFPK1* strain. (**C**) RT-PCR was used to identify transformants.

**Figure 4:**
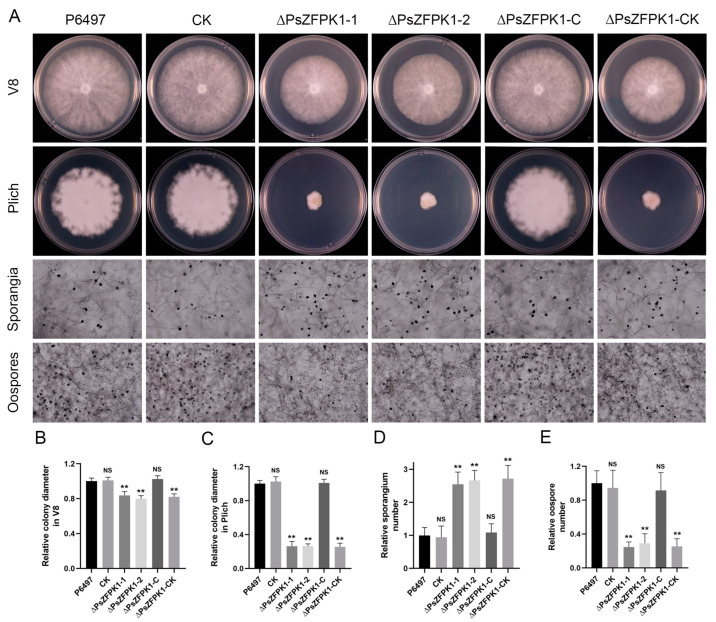
Phenotypic analysis of *PsZFPK1* gene knockout and complementation transformants. (**A**) Growth characteristics after 7 days on V8 medium and 9 days on Plich medium and microscopic visualization of the sporangia and oospores of the wild-type strain (P6497), unsuccessful *PsZFPK1* knockout (CK), *PsZFPK1* knockout (ΔPsZFPK1-1,2), complemented transformants (ΔPsZFPK1-C), and unsuccessfully complemented transformant (ΔPsZFPK1-CK). (**B**,**C**) Diameters of colonies were measured after 7 days on V8 medium (**B**) and 9 days on Plich medium (**C**), and relative colony diameters were calculated. (**D**,**E**) Numbers of sporangia (**D**) and oospores (**E**) relative to numbers in the wild-type strain P6497 (set at 1). All experiments were repeated three times. NS, not significant; asterisks indicate significant difference at *p* < 0.01 (**).

**Figure 5:**
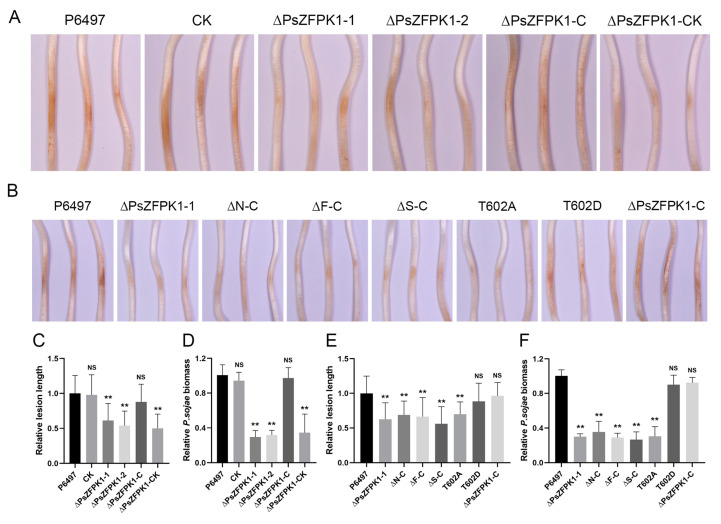
Virulence of *PsZFPK1* gene knockout, full-length complementation, truncated complementation, and T602A/D complementation transformants. (**A**,**B**) Lesions on soybean (Williams soybean cultivar) at 48 h post-inoculation (hpi) of 4-day-old etiolated hypocotyls with zoospores from (**A**), the wild-type strain (P6497), unsuccessful *PsZFPK1* knockout (CK), *PsZFPK1* knockout (ΔPsZFPK1-1,2), complemented transformants (ΔPsZFPK1-C), and unsuccessfully complemented transformant (ΔPsZFPK1-CK) and (**B**), the wild-type strain (P6497), *PsZFPK1* knockout (ΔPsZFPK1-1), N-terminal-truncated complemented transformant (ΔN-C), FYVE domain-truncated complemented transformant (ΔF-C), STKc domain-truncated complemented transformant (ΔS-C), and phosphorylation site mutation complemented transformants (T602A/D). (**C**,**E**) Lesion lengths on soybean plants were measured at 48 hpi, and relative lesion lengths were calculated. (**D**,**F**) Relative pathogen biomass in inoculated etiolated hypocotyls expressed as the ratio of *P. sojae* DNA to soybean DNA detected at 48 hpi relative to the P6497/soybean ratio (set at 1). All experiments were repeated three times. NS, not significant; asterisks indicate significant difference at *p* < 0.01 (**).

To investigate the role of *PsZFPK1*, we utilized a CRISPR/Cas9-mediated gene replacement system to generate knockout and complemented mutants. Genomic PCR analysis was then performed to screen the transformants. Here, we present the results of two representative *PsZFPK1* knockout mutants, ΔPsZFPK1-1 and ΔPsZFPK1-2, as well as one representative *PsZFPK1* complementary strain, ΔPsZFPK1-C. As controls, we used the wild-type strain (P6497) and two representative lines: CK, in which PsZFPK1 knockout was unsuccessful, and ΔPsZFPK1-CK, in which *PsZFPK1* was not successfully complemented (Figure 3A,B). RT-PCR was performed to confirm all mutants (Figure 3C). Compared with the WT and CK lines, the growth of the *PsZFPK1*-knockout mutants on nutrient-rich V8 plates was significantly reduced (Figure 4A). Furthermore, a more pronounced growth defect was observed on nutrient-poor Plich plates (Figure 4B). The *PsZFPK1*-knockout mutants exhibited significant increases in sporangium number (Figure 4C), consistent with the decreased *PsZFPK1* expression level observed at the asexual stage, while PsZFPK1 was also essential for producing oospores (Figure 4D). Phenotypic variation was restored in the ΔPsZFPK1-C transformant, which was obtained by CRISPR/Cas9-mediated in situ complementation. In addition, the lesion length produced by *PsZFPK1*-knockout transformants was significantly shorter than that produced by P6497 (Figure 5A,C,D). These results indicate that *PsZFPK1* is essential for the full virulence of *P. sojae*.

### 3.4. The N-Terminal Region, FYVE and STKc Domains, and T602 Phosphorylation Site Are Important for the Function of PsZFPK1

**Figure 6:**
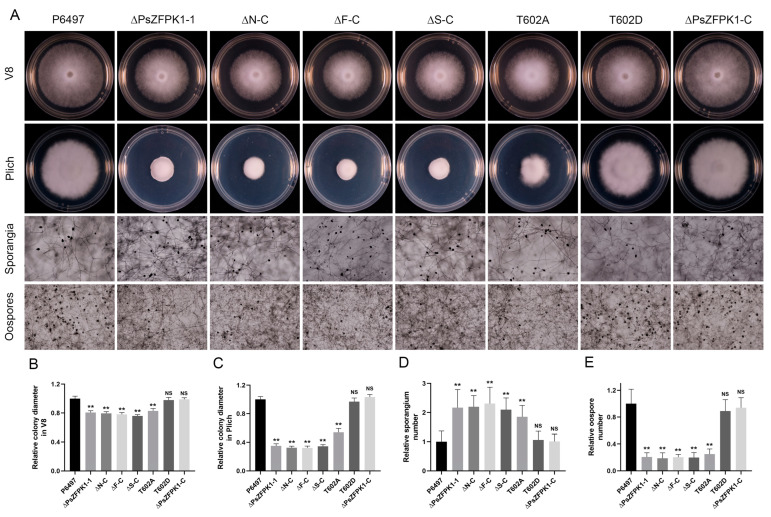
Phenotypic analysis of *PsZFPK1* gene knockout, full-length complementation, truncated complementation, and T602A/D complementation transformants. (**A**) Growth characteristics after 7 days on V8 medium and 9 days on Plich medium and microscopic visualization of sporangia and oospores of the wild-type strain (P6497), *PsZFPK1* knockout (ΔPsZFPK1-1), N-terminal-truncated complemented transformant (ΔN-C), FYVE domain-truncated complemented transformant (ΔF-C), STKc domain-truncated complemented transformant (ΔS-C), and phosphorylation site mutation complemented transformants (T602A/D). (**B**,**C**) Diameters of colonies were measured after 7 days on V8 medium (**B**) and 9 days on Plich medium (**C**), and relative colony diameters were calculated. (**D**,**E**) Numbers of sporangia (**D**) and oospores (**E**) relative to numbers in the wild-type strain P6497 (set at 1). All experiments were repeated three times. NS, not significant; asterisks indicate significant difference at *p* < 0.01 (**).

To identify the specific regions of PsZFPK1 responsible for its functional specificity, we generated donor plasmids with deletion of either the N-terminal region (2–321aa), FYVE domain, or STKc domain (Figure S2A) and transformed them into the *PsZFPK1* knockout mutant, ΔPsZFPK1-1. At least two transformants with the same phenotypes were obtained for each construct (one of each is shown in Figure S2B). Similar to the recipient PsZFPK1 knockout mutants, transformants with deletion of the N-terminal region, FYVE domain, or STKc domain exhibited defects in vegetative growth, sporangium formation, oospore production, and virulence (Figure 5B,E,F and Figure 6). These results indicate that the N-terminal region and the FYVE and STKc domains of PsZFPK1 are all necessary for these functions.

To investigate the significance of T602 phosphorylation, we introduced two mutations—an inactivated T602A and a constitutively activated phosphomimetic T602D—into the *PsZFPK1* mutant (Figure S2C). RT-PCR analysis confirmed the presence of all mutants (Figure S2D). The T602A transformant slightly restored the phenotypic defect of the *PsZFPK1* mutation. Intriguingly, the T602D transformant exhibited a phenotype similar to that of the complementary strain. These results suggest that phosphorylation of PsZFPK1 at T602 plays a crucial role in vegetative growth, sporangium formation, oospore production, and virulence (Figure 5B,E,F and Figure 6).

### 3.5. PsZFPK1 Is Not Necessary for the Stress Response

To determine the role of *PsZFPK1* in response to osmotic, oxidative, and cell wall integrity stresses, we investigated the inhibition ratio of *PsZFPK1*-knockout mutants in the presence of 0.25 M KCl, 0.5 M sorbitol, 2.5 mM/5 mM H_2_O_2_, and 1.2 µg/µL Congo red in V8 media. The results showed that the ΔPsZFPK1 mutants did not exhibit observable sensitivity to these stress-inducing agents (Figure S3). Therefore, it can be inferred that *PsZFPK1* is not essential for stress responses in *P. sojae*.

### 3.6. The Cytoplasmic Localization of PsZFPK1 Is Not Affected by the N-Terminal Region or the FYVE/STKc Domains

**Figure 7:**
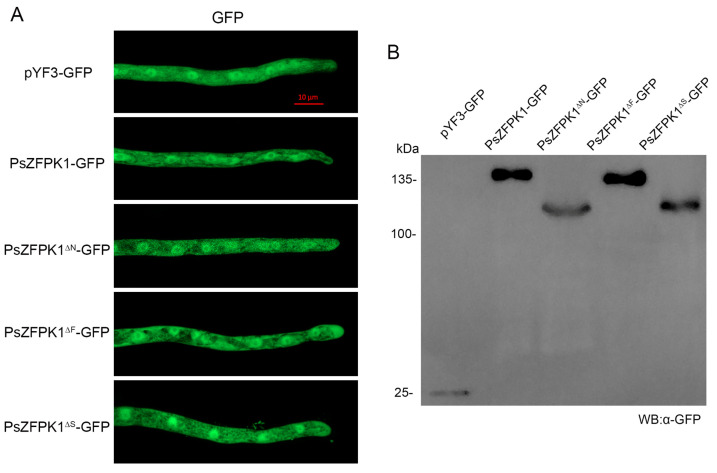
Subcellular localization of PsZFPK1 and truncated transformants lacking the N-terminal, FYVE domain, or STKc domain in the vegetative mycelia of *P. sojae*. (**A**) Images show the hyphae of transformants expressing an empty vector (pYF3-GFP) or full-length PsZFPK1 (PsZFPK1-GFP), or PsZFPK1 transformants lacking the N-terminus (PsZFPK1^ΔN^-GFP), FYVE domain (PsZFPK1^ΔF^-GFP), or STKc domain (PsZFPK1^ΔS^-GFP). The scale bar represents 10 µm. (**B**) Total proteins were extracted from fluorescence transformants and separated by SDS-PAGE. Immunoblots were performed using GFP antibody.

We used confocal microscopy to detect GFP-tagged PsZFPK1 protein and observe the hyphae of *P. sojae*. We observed localization of the GFP fluorescence in the cytoplasm, with the strongest fluorescence signal detected in the nucleus. This observation was consistent with the empty vector control pYF3-GFP transformants (Figure 7A). To investigate the impact of various regions on PsZFPK1 localization, we then examined GFP fluorescence transformants with deletions in the N-terminal region, FYVE domain, or STKc domain. All transformants showed cytoplasmic localization, consistent with the full-length PsZFPK1 protein (Figure 7A). These findings suggest that neither the N-terminal region, the FYVE domain, nor the STKc domain affects the cytoplasmic localization of PsZFPK1. Protein expression was confirmed by Western blot analysis (Figure 7B).

## 4. Discussion

The AGC protein kinase family is a group of eukaryotic serine/threonine kinases that regulate cell growth, metabolism, stress response, and pathogenicity in response to various external or internal signals [32,33,34]. Protein kinases are crucial targets for modern medicine development [35]. However, the lack of systematic research into the function and structure of protein kinases in plant pathogenic oomycetes hindered the development of inhibitors that could target protein kinases in these organisms. Interestingly, FYVE domain proteins are expanded in oomycete genomes, including that of *Phytophthora* spp. Moreover, a unique class of AGC protein kinase with FYVE as an accessory domain is found in protists, including *Phytophthora* spp. [19]. Our current results demonstrate that PsZFPK1, an AGC protein kinase with an FYVE domain, plays a critical role in vegetative growth, sporangium formation, oospore production, and virulence in *P. sojae*. The sequence specificity of these protein kinases indicates that they are potentially important fungicide targets.

Autophagy, as a stress response, plays a crucial role in the survival of eukaryotes [36,37]. Compared with the wild-type strain, P6497, *PsZFPK1*-knockout mutants exhibited significantly reduced growth on the Plich medium, a nitrogen starvation medium containing only 0.5 g yeast extract and 1 g asparagine per liter as a nitrogen source [28]. According to previous studies, nitrogen starvation can induce autophagy, which is essential for mycelial growth under nutrient-deprivation conditions [38,39]. These findings suggest that PsZFPK1 may be required for autophagy. Because of the relatively recent application of the CRISPR/Cas9-mediated gene knockout system in *P. sojae*, autophagy has rarely been studied in *P. sojae*. Nevertheless, 26 autophagy-related genes (ATGs) from the core autophagy machinery have been identified in *P. sojae*, and silencing of PsAtg6a in *P. sojae* was found to significantly reduce sporulation and pathogenicity [40]. The rapamycin-sensitive TORC1 protein complex negatively controls autophagy by inhibiting the activity of the protein kinase ATG1, which mediates an early activation step in the autophagic process [41,42]. Interestingly, the *PsZFPK1*-knockout mutants were less sensitive to rapamycin than P6497 (Figure S4). These results suggest that PsZFPK1 may regulate autophagy via the TORC1 signaling pathway.

The binding of FYVE proteins to PI3P, which is primarily located on early endosomes and multivesicular endosome vesicles, is mediated via the R(R/K)HHCR motif [43,44]. The R and H residues in the core R(R/K)HHCR motif provide critical hydrogen bonds to PI3P [45,46]. Generally, PI3P binding participates in the localization and function of FYVE proteins. For example, in *P. sojae*, deletion of the FYVE domain from PsFP1 weakened the vesicle-like subcellular localization of this protein [21]. In addition, PsFYVE1 specifically binds to PI3P based on conserved amino acids within the FYVE domain [22]. Interestingly, we identified the core motif of the FYVE domain in PsZFPK1 as RRIPCS, rather than R(R/K)HHCR, which suggests that PsZFPK1 may not bind specifically to PI3P. We also showed that full-length PsZFPK1 was cytoplasmic and that the FYVE domain did not affect protein localization. By contrast, the functional analysis indicated that the FYVE domain was necessary for the function of PsZFPK1. These results suggest that the biological function of the FYVE domain of PsZFPK1 may not depend on PI3P binding; however, the specific molecular mechanism remains to be resolved.

PsZFPK1 shares structural similarities with AKT and SGK3 protein kinases. Their accessory domains, the PH and PX domains, are major phosphoinositide-binding modules similar to the FYVE domain [47,48,49]. Previous studies have shown that PH domains can bind to PI(3,4)P2, PI(4,5)P2, and PI(3,4,5)P3 [50,51], while PX domains can bind to PI3P, PI4P, PI(3,4)P2, PI(4,5)P2, and PI(3,4,5)P3 [52,53]. The main phosphorylation sites in the AKT and SGK3 protein kinases are situated in the T-loop (activation loop) and HM (hydrophobic motif) region [8,10], corresponding to positions 602 and 766 of PsZFPK1, respectively. We showed that the residue at site 766 of PsZFPK1 was alanine. Phosphorylation sites corresponding to site 602 are commonly phosphorylated by the protein kinase PDK1 [54,55]. Our data demonstrated that the T602 phosphorylation site of PsZFPK1 was required for its function, suggesting that PsZFPK1 may function as a downstream effector of PDK1. Moreover, while the activation of AKT and SGK3 requires their translocation to the cell membrane and binding to phosphatidylinositol via the PH or PX domains, the FYVE domain of PsZFPK1 did not affect its localization, indicating that PsZFPK1 activation may not depend on these processes.

Overall, there is a dearth of studies on proteins containing both FYVE and STKc domains in plant pathogenic oomycetes. Our study demonstrates the crucial role of PsZFPK1 in vegetative growth, sporangium formation, oospore production, and virulence in *P. sojae*. Future research will focus on the functional analysis of the FYVE domain and the exploration of the downstream effectors of PsZFPK1. Our findings will enhance the understanding of proteins with FYVE and STKc domains and bridge the gap in research on the function of these proteins in oomycetes.

## 5. Conclusions

In summary, we characterized the functions of a newly identified FYVE domain-containing protein kinase PsZFPK1 in *P. sojae* with a CRISPR/Cas9 gene knockout system. Our results demonstrate that PsZFPK1 plays a crucial role in vegetative growth, sporangium formation, oospore production, and virulence. Our findings provide a fundamental research basis for further exploring the molecular regulation mechanism of PsZFPK1 in *P. sojae*. Our results will enrich the understanding of the FYVE domain-containing protein kinase and fill the international gap in the research on the functions of FYVE domain-containing protein kinase in oomycetes.

## Data Availability

Not applicable.

## Supplementary Materials

The following supporting information can be downloaded at https://www.mdpi.com/article/10.3390/jof9070709/s1. Figure S1: Standard curves of qRT-PCR primers for *PsActin* and *PsZFPK1*; Figure S2: Lacking the N-terminal, FYVE domain, or STKc domain and the T602A/D phosphorylation site mutation complemented transformants’ confirmation; Figure S3: Stress response of *PsZFPK1* gene knockout and complementation transformants; Figure S4: Sensitivity of *PsZFPK1* knockout transformants to rapamycin; Table S1: sgRNA and Primers used in this study.
