# Supplementary figures and images for: A Novel FYVE Domain-Containing Protein Kinase, PsZFPK1, Plays a Critical Role in Vegetative Growth, Sporangium Formation, Oospore Production, and Virulence in Phytophthora sojae
Source: J Fungi (Basel). 2023 Jun 28;9(7):709. doi: 10.3390/jof9070709 (PMC10381902; doi:10.3390/jof9070709)

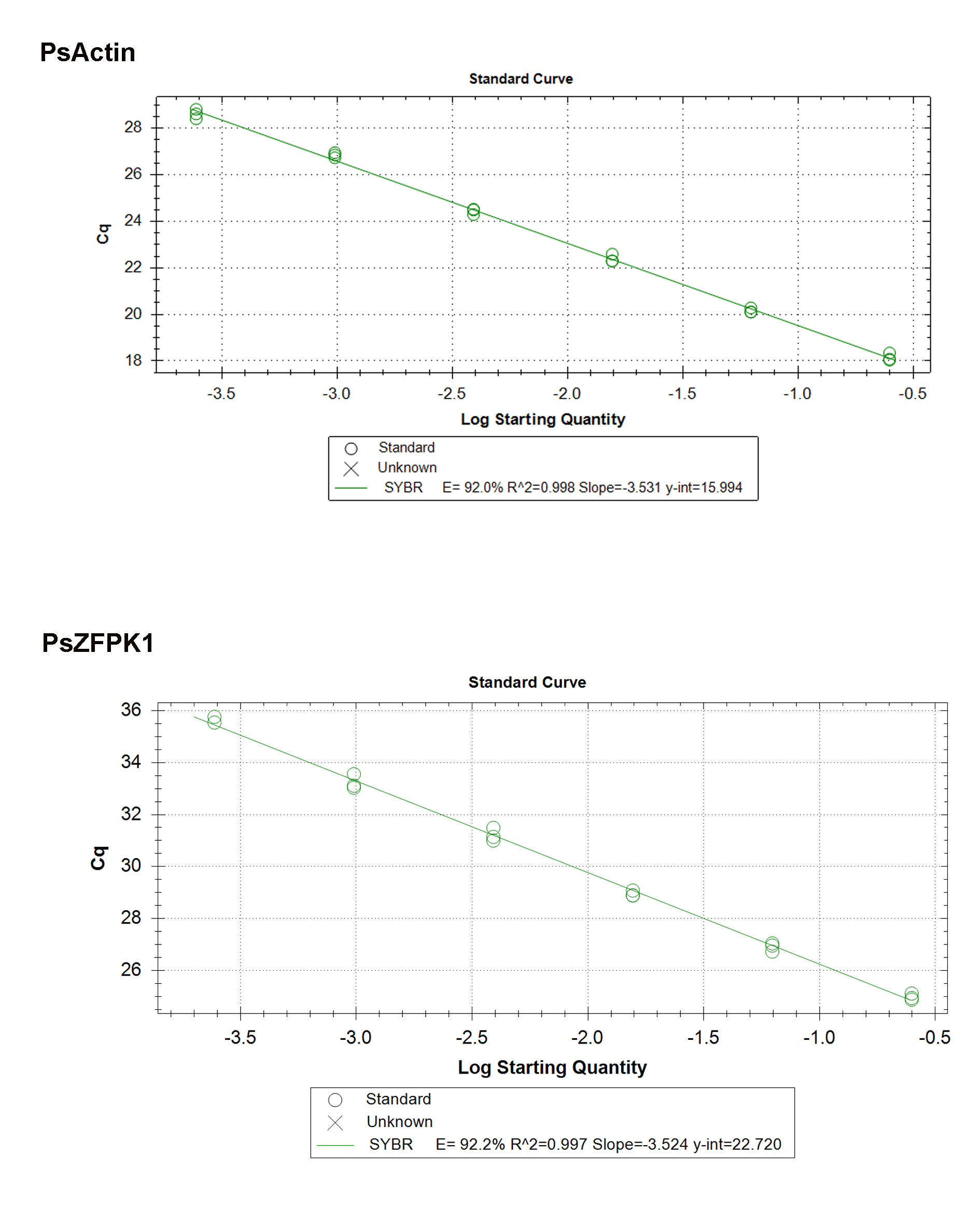

Supplement: Supplementary file 1 [file jof-09-00709-s001.zip › Fig S1.tif]

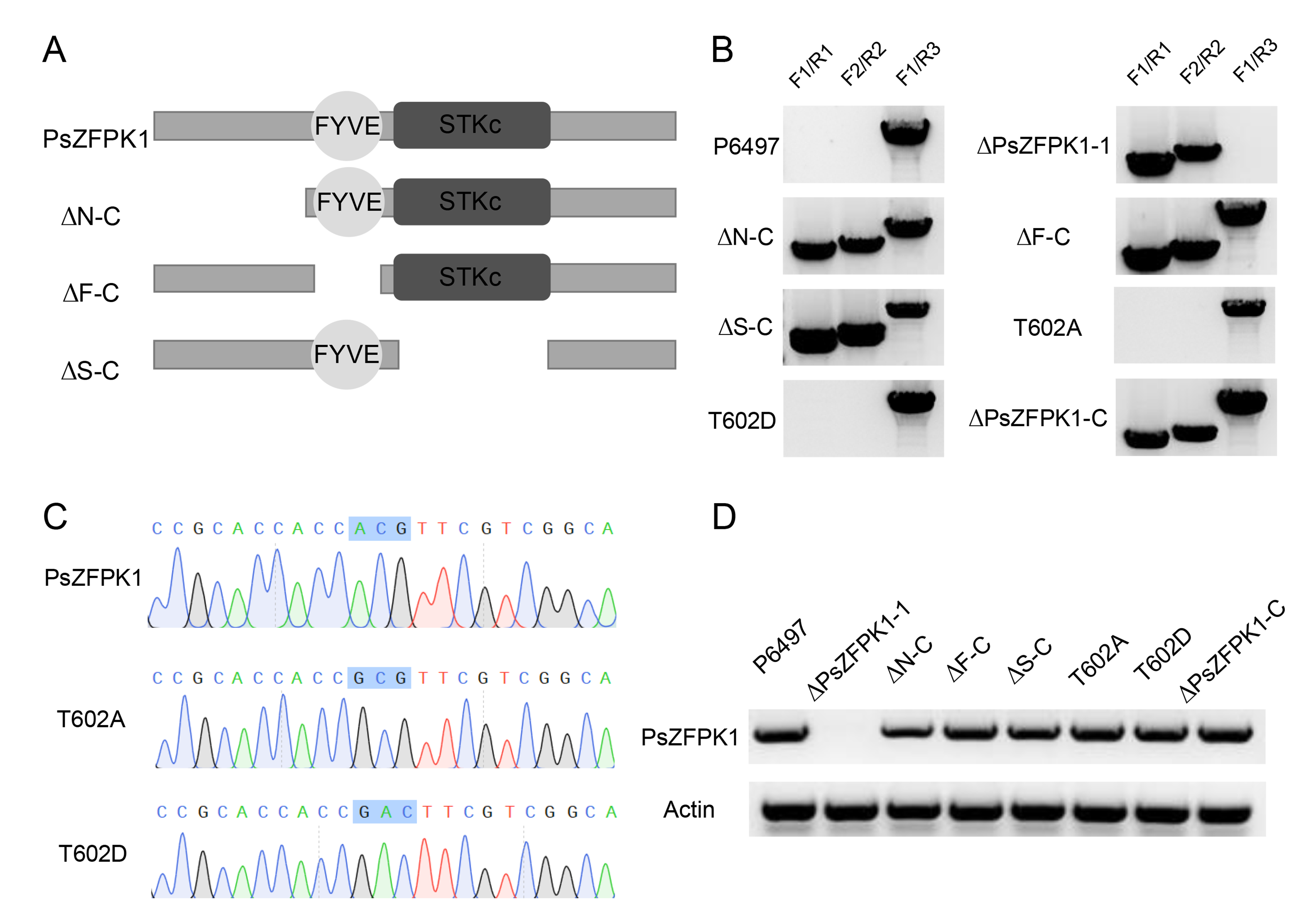

Supplement: Supplementary file 1 [file jof-09-00709-s001.zip › Fig S2.tif]

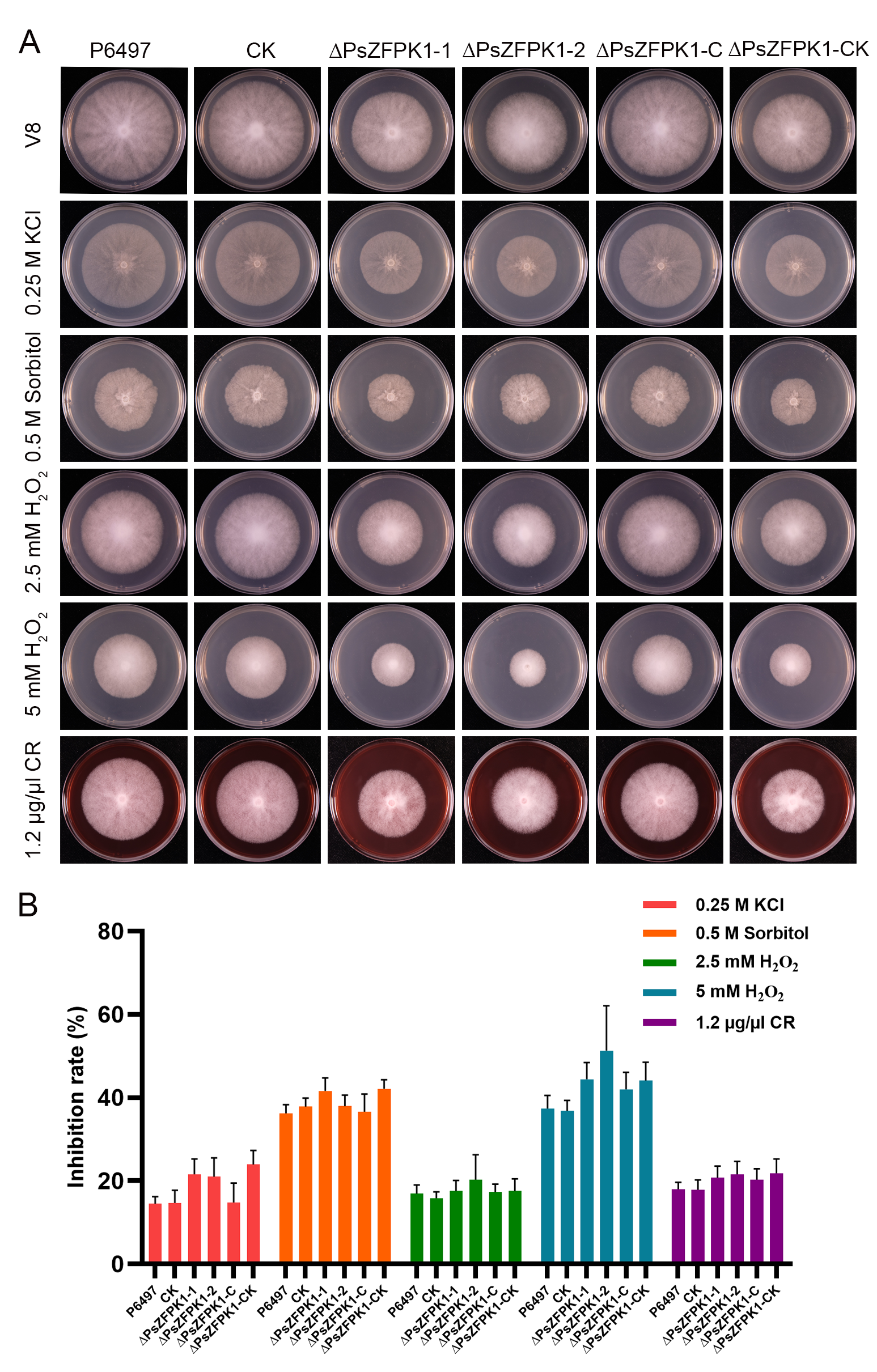

Supplement: Supplementary file 1 [file jof-09-00709-s001.zip › Fig S3.tif]

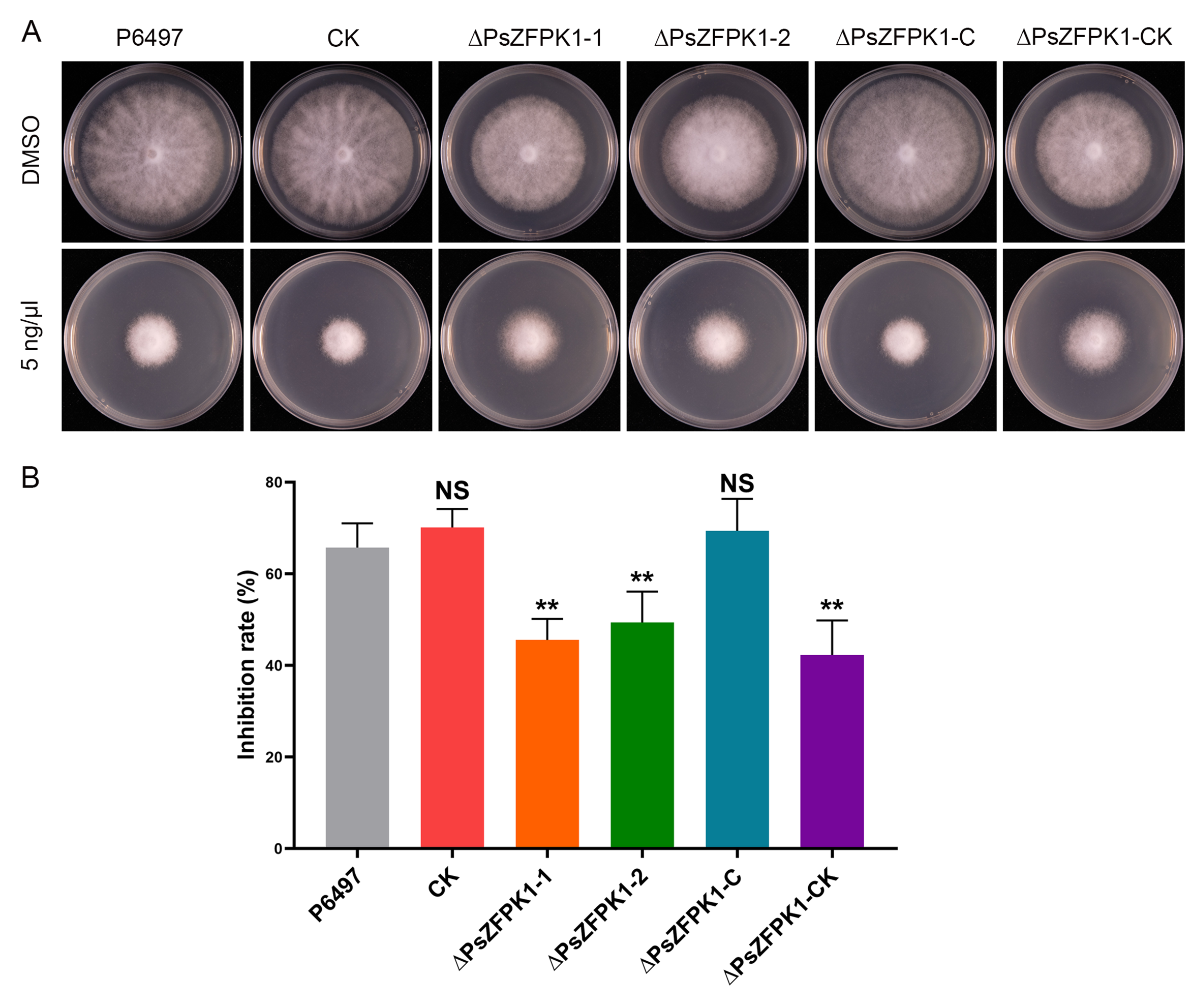

Supplement: Supplementary file 1 [file jof-09-00709-s001.zip › Fig S4.tif]
